# Adjuvant Effects Elicited by Novel Oligosaccharide Variants of Detoxified Meningococcal Lipopolysaccharides on *Neisseria meningitidis* Recombinant PorA Protein: A Comparison in Mice

**DOI:** 10.1371/journal.pone.0115713

**Published:** 2014-12-29

**Authors:** Ojas H. Mehta, Gunnstein Norheim, J . Claire Hoe, Christine S. Rollier, Jerry C. Nagaputra, Katherine Makepeace, Muhammad Saleem, Hannah Chan, David J. P. Ferguson, Claire Jones, Manish Sadarangani, Derek W. Hood, Ian Feavers, Jeremy P. Derrick, Andrew J. Pollard, E . Richard Moxon

**Affiliations:** 1 Oxford Vaccine Group, Department of Paediatrics, University of Oxford, and the NIHR Biomedical Research Centre, Churchill Hospital, Headington, Oxford, OX3 7LE, United Kingdom; 2 Faculty of Life Sciences, The University of Manchester, Michael Smith Building, Oxford Road, Manchester, M139PT, United Kingdom; 3 Division of Bacteriology, National Institute of Biological Standards and Control, Blanche Lane, South Mimms, Potters Bar, Hertfordshire, EN6 3OG, United Kingdom; 4 Nuffield Department of Clinical Laboratory Sciences, University of Oxford, Oxford, United Kingdom; 5 Department of Paediatrics, Children's Hospital (John Radcliffe), Headley Way, Headington, Oxford, OX3 9DU, United Kingdom; 6 The NIHR Oxford Biomedical Research Centre, Centre for Clinical Vaccinology and Tropical Medicine, Churchill Hospital, Headington, Oxford, OX3 7LE, United Kingdom; Universidad Nacional de La Plata, Argentina

## Abstract

*Neisseria meningitidis* lipopolysaccharide (LPS) has adjuvant properties that can be exploited to assist vaccine immunogenicity. The modified penta-acylated LPS retains the adjuvant properties of hexa-acylated LPS but has a reduced toxicity profile. In this study we investigated whether two modified glycoform structures (LgtE and IcsB) of detoxified penta-acylated LPS exhibited differential adjuvant properties when formulated as native outer membrane vesicles (nOMVs) as compared to the previously described LgtB variant. Detoxified penta-acylated LPS was obtained by disruption of the *lpxL1* gene (LpxL1 LPS), and three different glycoforms were obtained by disruption of the *lgtB*, *lgtE* or *icsB* genes respectively. Mice (*mus musculus*) were immunized with a recombinant PorA P1.7-2,4 (rPorA) protein co-administered with different nOMVs (containing a different PorA serosubtype P1.7,16), each of which expressed one of the three penta-acylated LPS glycoforms. All nOMVs induced IgG responses against the rPorA, but the nOMVs containing the penta-acylated LgtB-LpxL1 LPS glycoform induced significantly greater bactericidal activity compared to the other nOMVs or when the adjuvant was Alhydrogel. Compared to LgtE or IcsB LPS glycoforms, these data support the use of nOMVs containing detoxified, modified LgtB-LpxL1 LPS as a potential adjuvant for future meningococcal protein vaccines.

## Introduction

*Neisseria meningitidis* is a major cause of meningitis and septicaemia globally. Efficient polysaccharide-protein glycoconjugate vaccines are available against 4 (A, C, W and Y) of the 5 major disease-causing capsular groups, but not for strains of capsular group B [1]. Vaccines containing outer membrane vesicles (OMVs) have been developed, including one licensed vaccine containing OMVs mixed with recombinant outer membrane proteins [1]. OMVs bear a number of outer membrane antigens within a vesicular membrane and are immunogenic, eliciting protection against homologous strains, in particular those expressing the same variant of the immunodominant protein PorA [2]. However, OMVs are unable to induce persisting immune responses in infants and are reactogenic [3], [4], prompting research into improvements and molecular modifications of OMVs [5].

Lipopolysaccharide (LPS) is a component of the meningococcal outer membrane. It is largely responsible for stimulating the destructive inflammatory cascade during invasive disease, and is partially removed from OMV-based vaccines by detergent extraction whereby the relative LPS content is lowered from 20–25% to 5–7% [6], enabling safe administration in humans [7]. LPS consists of a hydrophobic lipid A component (endotoxin) anchored within the outer membrane and a hydrophilic oligosaccharide extension [8]. Lipid A is synthesized through a series of acylation steps concluding with the addition of a sixth 12-carbon acyl chain by the enzyme LpxL1 [9]. Altered forms of LPS have been proposed as potential vaccine adjuvants [10], [11], [12], [13], [14]. Detoxifying LPS by disruption of *lpxL1* (LpxL1 LPS) results in expression of penta-acylated lipid A, which has significantly lower toxicity than the usual hexa-acylated form, but retains the adjuvant properties [9]. Inactivation of *lpxL1* allows native OMVs (nOMVs) containing high levels of penta-acylated LPS to be safely administered to humans [7]. This has the additional advantage of retaining low molecular weight antigens such as factor-H binding protein (fHbp), which contribute to enhanced vaccine immunogenicity [7], and would otherwise be removed during detergent-dependent detoxification processes. Introducing an *lpxL1* mutation is therefore an attractive approach that preserves the adjuvant effect of LPS whilst minimizing its toxicity in OMVs. LPS exhibits its biological effects through the activation of Toll-like receptor 4 (TLR-4) on dendritic cells (DC) and other cell types. LPS glycoforms with varied oligosaccharide lengths can modulate DC activation and down-stream signalling, thus altering T-cell behaviour [15], [16]. The adjuvant effect of a modified glycoform of lipopolysaccharide (LPS) (LgtB-LpxL1) on immune responses of mice to vaccination with meningococcal protein, tetanus toxoid, or meningococcal serogroup C capsular polysaccharide was recently demonstrated and compared to the responses induced by the non-modified glycoform Lpxl1 [14].

This study investigates the capability of two different LPS oligosaccharide variants of penta-acylated LpxL1 LPS, as compared to the previously identified LgtB-LpxL1, to alter bactericidal immune responses. To this end, we have disrupted the genes encoding the glycosyltransferases LgtB, LgtE and IcsB in different strains of *N. meningitidis*, each resulting in the sequential shortening of the LPS by one monosaccharide. We investigated the adjuvant properties of these LPS structures in mice when delivered in the form of nOMVs in combination with the recombinant protein rPorA P1.7-2,4.

## Materials and Methods

### Bacterial strains and growth conditions

*E. coli* strains DH5α (Invitrogen) and NovaBlue (Merck) were cultured at 37°C on Luria-Bertoni (LB) media. *N. meningitidis* strains used in this study are summarized in Table 1 and included H44/76 (B:15:P1.7,16:L3,7,9), mutants derived from strain MC58 (B:15:P1.7,16b, with disrupted *lgtB* [17] or *lgtE* [17] genes) and H44/76 (disrupted *icsB* gene [18]), and the disease isolates 91/40 (B:4:P7-2,4) and BZ198 (B:NT:P1.7-2,4). Meningococci were cultured at 37°C in 5% CO_2_ (v/v) in modified liquid Frantz medium [19] or solid brain heart infusion (BHI) media (Merck) supplemented with Levinthal's reagent (10% v/v).

**Table 1:** Genotype of strains used and constructed.

Strains	Description
*E. coli* DH5α	F- φ80*lac*ZΔM15 Δ(*lac*ZYA-*arg*F)U169 *rec*A1 *end*A1 *hsd*R17(rk-, mk+) *pho*A *sup*E44 *thi*-1 *gyr*A96 *rel*A1 *ton*A
*E. coli* NovaBlue	endA1 hsdR17(rK12– mK12+) supE44 thi-1 recA1 gyrA96 relA1 lac F′ [proA+B+ lacIqZΔM15::Tn10 (TcR)]
*E. coli* BL21(DE3)	F– ompT hsdSB(rB– mB–) gal dcm (DE3)
H44/76	As wildtype; http://pubmlst.org
MC58-LgtB	*lgtB* disrupted with a kanamycin resistance cassette in strain MC58
MC58-LgtE	*lgtE* disrupted with a kanamycin resistance cassette in strain MC58
H44/76-IcsB	*icsB* disrupted with a kanamycin resistance cassette in strain H44/76
BZ198	As wildtype; http://pubmlst.org
91/40	As wildtype; http://pubmlst.org
H44/76-LgtB	*lgtB* disrupted with a kanamycin resistance cassette
H44/76-LgtE	*lgtE* disrupted with a kanamycin resistance cassette
H44/76-LgtB-LpxL1	*lgtB* disrupted with a kanamycin resistance cassette, *lpxL1* disrupted with a tetracycline resistance cassette
H44/76-LgtE-LpxL1	*lgtE* disrupted with a kanamycin resistance cassette, *lpxL1* disrupted with a tetracycline resistance cassette
H44/76-IcsB-LpxL1	*icsB* disrupted with a kanamycin resistance cassette, *lpxL1* disrupted with a tetracycline resistance cassette

### Construction of meningococcal mutants expressing truncated and detoxified LPS

The LPS glycosyltransferase genes in *N. meningitidis* strain H44/76 were disrupted by transformation with chromosomal DNA from previously constructed mutant strains with disrupted, *lgtE* [17] and *icsB* [18]. Disruption of *lgtB* was described previously [14]. Meningococcal genomic DNA was purified with a QIAamp DNA Mini Kit (Qiagen). A 1 µl loopful of overnight plate grown *N. meningitidis* H44/76 was transformed with 15–50 ng of DNA, as described previously [20]. Transformants were obtained following growth on selective media (kanamycin 100 µg/ml). Mutation was confirmed by PCR analyses using purified chromosomal DNA and through analysis of LPS profiles using 16.5% acrylamide Tris-Tricine gels run at 30 mA and 4°C for 18 hours [21] and silver stained (GE Healthcare) according to the manufacturers' instructions. Disuption of *lpxL1* gene was performed as described previously [14].

### Construction, expression and purification of rPorA P1.7-2,4

Recombinant rPorA P1.7-2,4 was prepared by cloning *porA P1.7-2,4* into plasmid apET30-ekLIC and transforming into *E. coli* strain T7 Express (New England Biolabs, Hitchin, UK). Bacteria were harvested, disrupted by sonication, inclusion bodies isolated as previously described [22] then resuspended in Tris buffer (10 mM, pH 7.5), EDTA (1 mM) and urea (8 M). Following centrifugation at 14000×*g* for 20 minutes to remove cell debris, the supernatant was added in a 1∶1 ratio whilst stirring rapidly to a buffer comprising Tris (20 mM, pH 7.9), NaCl (1 M) and zwittergent 3–14 (ZW 3–14) (2% w/v) before being dialyzed against Tris (20 mM, pH 7.9), NaCl (0.5 M) and ZW3-14 (0.05% w/v) for two periods of 6–8 hours at 4°C. The resulting solution was passed through a 0.45 µm filter then applied to a HISTrap HP column (GE Healthcare) in Tris (20 mM, pH 7.9), NaCl (0.5 M), lauryldimethylamine oxide (0.1% v/v) and imidazole (10 mM). The column was washed with the same buffer containing 40 mM imidazole. 500 mM imidazole was used to elute rPorA, which was then dialyzed against Tris (10 mM, pH 7.9), NaCl (150 mM) and ZW 3–14 (0.05% w/v). A circular dichroism spectrum was run using a JASCO J-10 spectrometer with a 0.05 cm path length quartz cell, to confirm the folded state of the rPorA preparation.

### Production of Outer Membrane Vesicles

Extraction of nOMVs were performed as previously described [14]. Production of the nOMVs was performed five times for each strain, and the protein content of the nOMVs was quantified each time using a micro Lowry assay [23] (Sigma). The protein antigen profiles were assessed for each production lot by sodium dodecyl sulphate – polyacrylamide gel electrophoresis (SDS-PAGE) with coomassie brilliant blue staining. LPS profiles were assessed using 16.5% acrylamide Tris-Tricine gels as described above. The structure of OMVs was characterized by transmission electron microscopy using methyl tungstate negative staining [24]. The size distribution was assessed by nanoparticle tracking analysis (NTA) using a Nanosight NS500 system (NanoSight). The Brownian motion was tracked for each particle, allowing calculation of the size through the application of the Stokes-Einstein equation.

### Effect of of different LPS glycoforms on reactogenicity

The effect on reactogenicity induced by the modifications in the LPS of the different variants was analyzed by standard proinflammatory cytokine production by human whole blood stimulation assay, as described previously [25]. Quantification of IL-6 in the cell supernatant was performed by Human IL-6 ELISA kit (Invitrogen) according to the manufacturer's instructions.

### Murine immunizations

This study was carried out in strict accordance with the regulations of the UK Animals (Scientific Procedures) Act 1986. The protocol was approved by the local ethics committee on animal experiments at NIBSC (Home Office Project Licence Number 80/2157). All immunizations were performed under anesthesia, and all efforts were made to minimize suffering. Samples were obtained following terminal general anaesthesia. Mice were housed in groups in a specific pathogen free facility, under controlled light/dark cycle and temperature housing, with access to food and water *ad libitum* and with environmental enrichment, under the control of a named veterinary surgeon. Groups of 10 healthy inbred 6–8 week old NIH/OlaHsd female mice (Harlan UK) weighing between 18–22 g were immunized subcutaneously on days 0 and 28 and terminally bled on day 42. Mice were immunized with 5.0 µg of purified rPorA P1.7-2,4 with or without 330 µg Alhydrogel or 2.5 µg of nOMVs from the *lpxL1* mutant strains (Table 2). Control groups of mice were immunized with Alhydrogel or nOMVs alone. A power calculation was performed using data from a group of mice immunized with rPorA + Alum (mean test value titer 165, standard deviation 99.9). Ten animals per group provide 99.9% statistical power at an Alpha error level of 5% to detect a 2-fold difference with a two-tail test. No adverse events were observed during the experiments.

**Table 2:** Immunization schedules.

Group	Antigen	Adjuvant	Adjuvant dose
1	5 µg rPorA P1.7-2, 4		
2	5 µg rPorA P1.7-2, 4	Alhydrogel	330 µg
3	5 µg rPorA P1.7-2, 4	nOMV: H44/76-LgtB-LpxL1	2.5 µg
4	5 µg rPorA P1.7-2, 4	nOMV: H44/76-IcsB-LpxL1	2.5 µg
5	5 µg rPorA P1.7-2, 4	nOMV: H44/76-LgtE-LpxL1	2.5 µg
6		nOMV: H44/76-LgtB-LpxL1	2.5 µg
7		nOMV: H44/76-IcsB-LpxL1	2.5 µg
8		nOMV: H44/76-LgtE-LpxL1	2.5 µg
9		Alhydrogel	330 µg

### Analysis of serum antibody responses by ELISA

An OMV enzyme-linked immunosorbent assay (ELISA) to quantify specific IgG antibodies raised in mice was adapted from that of Rosenqvist et al. [26]. Maxisorb 96-well microtiter plates (Nunc) were coated overnight at 37°C with 5 µg/ml of BZ198 nOMVs. Plates were washed five times with 150 mM NaCl, 0.05% (v/v) Tween-20 before serial double-dilutions of sera in PBS, 0.5% BSA, 0.05% (v/v) Tween-20 were added. After incubation for 2 hours at 37°C, the plates were washed and incubated for a further 2 hours at 37°C with alkaline-phosphatase anti-mouse IgG conjugate (Sigma) diluted 1∶5000. The plates were then washed and incubated for 25 min at room temperature with the substrate 4-nitrophenyl phosphate disodium salt hexahydrate (pNpp) (Sigma) diluted in substrate dilution buffer (Zymed Laboratories Inc) as per the manufacturer's instructions. The colour reaction was stopped with 5 µl of 3 M sodium hydroxide. Optical densities were read at 405 nm with a 620 nm reference.

Anti-PorA IgG titers were quantified as as described previously [14].

An internal serum standard was prepared by pooling sera identified as high responders. A standard curve was fit using a four-parametric model as described by Plikaytis et al. [27]. Samples that did not show OD values within the range of the standard curve at dilutions of 1∶125 or greater were assigned an arbitrary concentration of 0.097 Units/ml as calculated according to WHO guidelines [28].

### Serum Bactericidal Assay

A serum bactericidal activity (SBA) assay was adapted from methods previously described [29] and was used to assess bactericidal activity against *N. meningitidis* strain BZ198, using pooled murine sera and baby rabbit complement (lot 11330, Pel-Freez Biologicals, Rogers, AR) to a final concentration of 25%, as described previously [14]. The serum bactericidal titre was measured as the reciprocal of the last serum dilution that resulted in greater than 50% killing of the viable count (T60). The T60 was calculated from control wells containing bacteria and heat-inactivated sera. Other controls included bacteria alone and bacteria plus complement.

### Statistical analyses

Antibody titres against BZ198 nOMVs and rPorA P1.7-2,4 were log_10_ transformed and analysed with SPSS Statistics 17.0 (SPSS Inc) and GraphPad Prism 5 (GraphPad Software, Inc). Analysis of variances (ANOVA) was used for statistical evaluation of data.

## Results

### Construction of H44/76 double mutant strains

**Figure 1:**
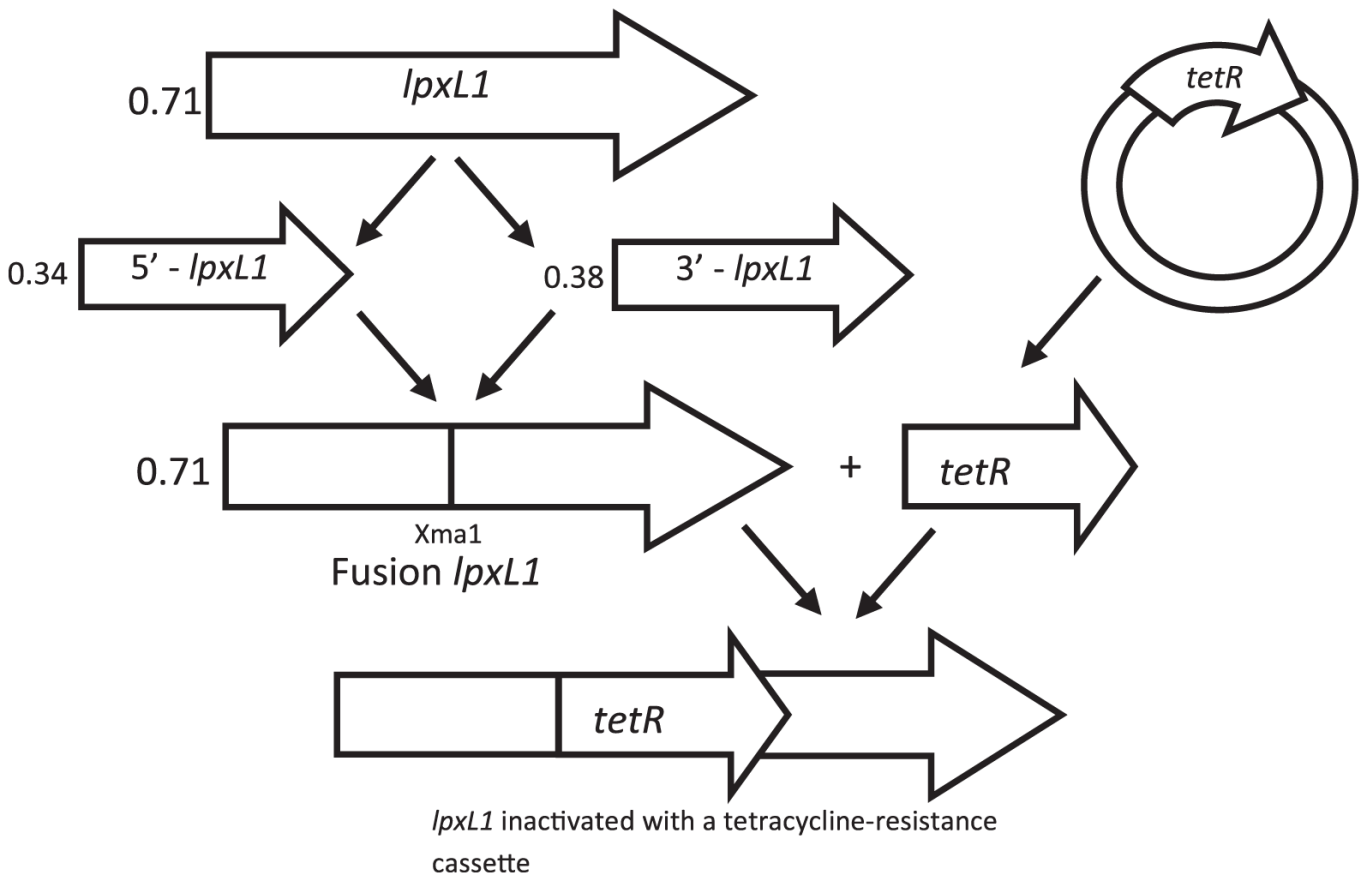
Schematic representation of the construction strategy showing PCR products and plasmid construction. Separate amplification of the 5′ and 3′ ends of the MC58 *lpxL1* gene, introduction of a novel *XmaI* restriction enzyme site by fusion PCR, and introduction of the *tetR* resistance gene to disrupt lpxL1 with cloning of the resulting *ΔlpxL1* into pCR2 plasmid, which was then used for *N. meningitidis* strain transformation. The numbers indicate the size of the fragments in Kbp.

**Figure 2:**
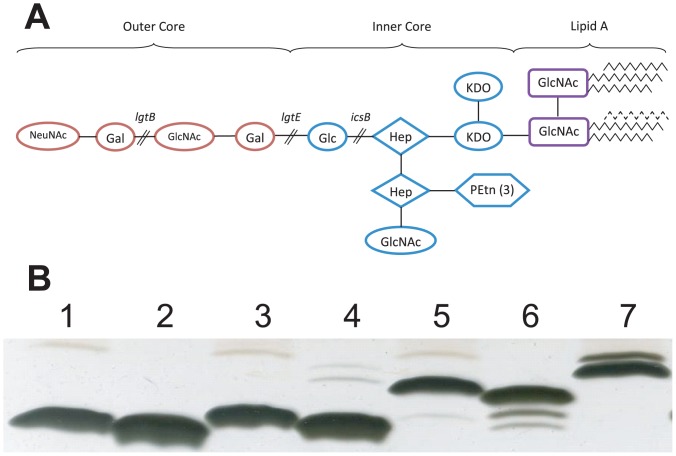
LPS structure of meningococcal strain H44/76. (A) The genes disrupted in this study are indicated and this resulted in truncated glycoforms of different lengths. Disruption of *lpxL1* prevented the addition of the final acyl chain which is indicated by the dotted line. Key: Gal – galactose, GlcNAc – N-acetyl glucosamine, Glc – glucose, Hep – heptose, PEtn – phosphoethanolamine, NeuNAc – *N*-acetyl neuraminic acid, KDO – 2-keto-3-deoxyoctulosonic acid. (B) LPS profiles of H44/76 mutants obtained by electrophoresis using 16.5% acrylamide Tris-Tricine gels and silver staining. LPS from cell lysates from strains (1) H44/76-IcsB, (3) H44/76-LgtE and (5) H44/76-LgtB migrate slightly slower than LPS from OMVs of strains (2) H44/76-IcsB-LpxL1, (4) H44/76-LgtE-LpxL1 (6) H44/76-LgtB-LpxL1, as the latter each lack an acyl chain. LPS from strain H44/76 (7) was run for comparison.

To examine the effect of LPS oligosaccharide length on the adjuvant properties of OMVs, mutants with disrupted *lgtB, lgtE* and *icsB* genes were constructed in the prototypic *N. meningitidis* serogroup B strain H44/76 background. H4476 was transformed with chromosomal DNA from the appropriate, characterised, LPS mutants (*lgtB, lgtE* and *icsB*) of strain MC58 (Fig. 1). The expected genetic disruption in the mutants was confirmed through growth on selective media and PCR analysis (data not shown). The expected LPS phenotypes of the single and double mutant strains, described in Fig. 2A, were consistently confirmed on five independent occasions by TSDS-PAGE of cell lysates for the single mutants or nOMVs obtained from the double mutants, as shown in Fig. 2B for the strains subsequently used for murine immunization (Fig. 2B).

### Production and characterisation of nOMVs

**Figure 3:**
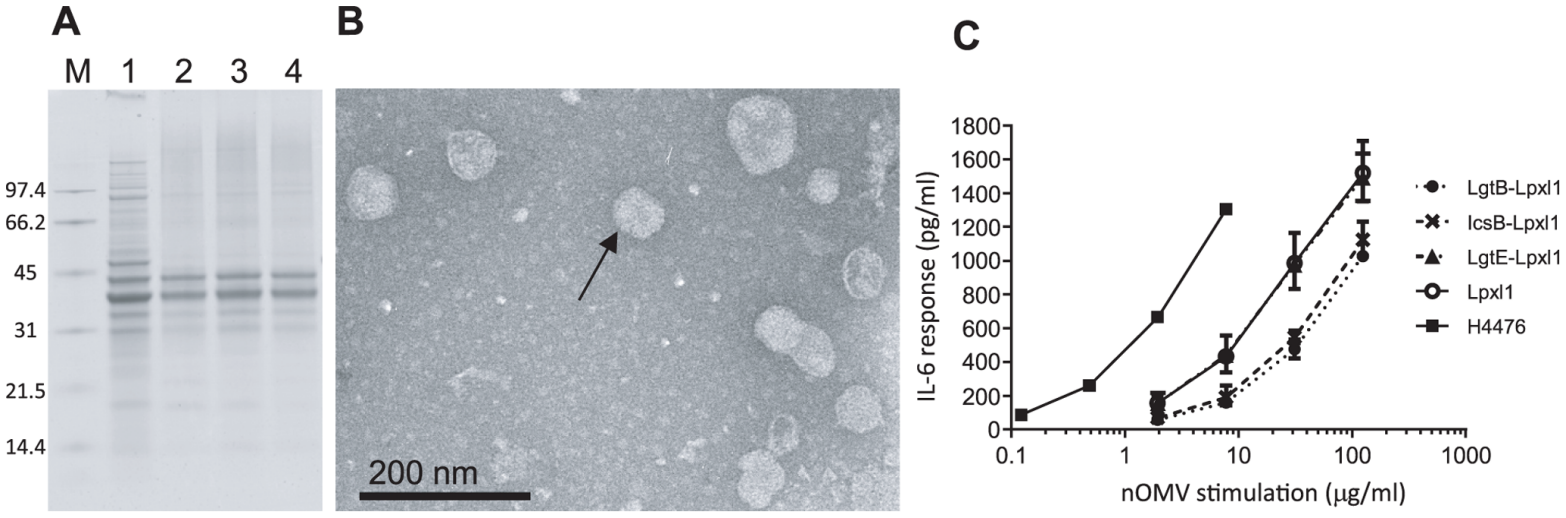
Characterization of nOMVs. (A) The protein content was analysed by SDS PAGE of OMVs obtained from 1: H44/76-Lpxl1, 2: H44/76-IcsB-Lpxl1, 3: H44/76-LgtB-Lpxl1, 4: H44/76-LgtE-Lpxl1 strains. M: molecular weight marker, with sizes indicated on the left in kDa (B) Electron micrograph illustrating H44/76-LgtB-LpxL1 nOMVs (arrow). OMVs obtained from other strains had equivalent phenotypes (not shown). (C) Effect of nOMVs on IL-6 production by human blood. The average results of *in vitro* whole-blood stimulation assay comparing the responses to nOMVs prepared from the strains expressing LPSs with different structures as indicated in the legend are represented (geometric means of two measurements, except for the positive control H44/76, with error bars representing the 95% confidence intervals).

nOMVs were extracted from strains H44/76-LgtB-LpxL1, H44/76-LgtE-LpxL1 and H44/76-IcsB-LpxL1 on five occasions, and the resulting nOMVs were characterized by SDS-PAGE, TSDS-PAGE and electron microscopy. The results from the production subsequently used for murine immunization are representative of the 5 production rounds and are shown below. TSDS-PAGE demonstrated differences in molecular weight of the LPS between the nOMVs that corresponded with the predicted alterations to each LPS glycoform, although a reduction, interpreted to be minor, in molecular weight of LPS was observed for each *lpxL1* mutant when compared to the respective parent strain (Fig. 2B). SDS-PAGE confirmed that each of the three oligosaccharide-modified nOMV preparations had similar protein contents compared to those from a *lpxL1* single mutant (Fig. 3A). The nOMVs visualized by electron microscopy, and the particle size distribution (performed by NTA) confirmed that samples contained mostly intact vesicles with some cell debris, with a range in size from 30 nm to 150 nm (Fig. 3B). Their appearance was similar to those previously obtained by us and others [30].

Since reactogenicity and adjuvant capacity are related, we experimentally analyzed the effect on reactogenicity induced by the modifications in the LPS of the different nOMVs. IL-6 was quantified in supernatant of blood samples cultured with the different nOMVs (Fig. 3D). The pentacylated LPS obtained by Lpxl1 mutation decreases IL-6 production as compared to hexaacylated LPS in H44/76 nOMVs, as expected, (2) Lpxl1 and Lpxl1-LgtE nOMVs induced similar IL-6 production, and (3) Lpxl1-IcsB and Lpxl1-LgtB nOMVs induced lower IL-6 production as compared to the 2 other Lpxl1 variants.

### Characterisation of purified recombinant rPorA P1.7-2,4

**Figure 4:**
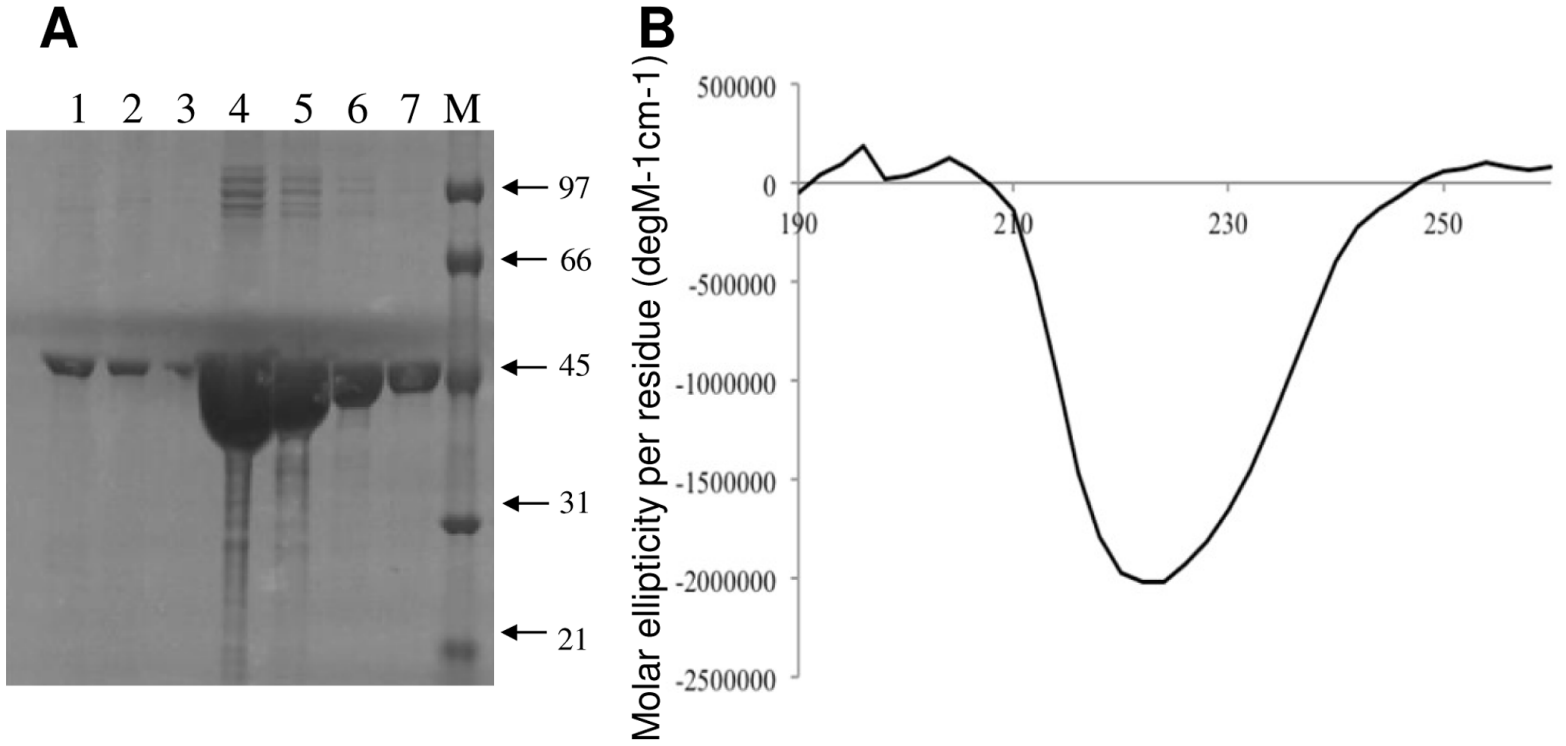
Characterization of recombinant rPorA protein. (A) rPorA protein was purified on a metal chelate affinity column, the SDS-PAGE patterns of elution fractions 1 to 7 are shown. M indicates the molecular weight marker. (B) Circular dichroism spectrum indicating correctly folded rPorA P1.7-2,4.

The modified nOMVs express PorA P1.7,16 (strain H44/76). Heterologous recombinant rPorA P1.7-2,4 was expressed in *E. coli* and purified to greater than 90% (Fig. 4A). Circular dichroism spectrometry confirmed that rPorA P1.7-2,4 was correctly folded (Fig. 4B). The spectrum was similar to that reported previously for PorA purified from OMVs [31]. The expression and purification of recombinant PorA was carried out on at least six separate occasions, with similar results obtained for all runs.

### The three nOMVs elicit antibody responses to rPorA P1.7-2,4

**Figure 5:**
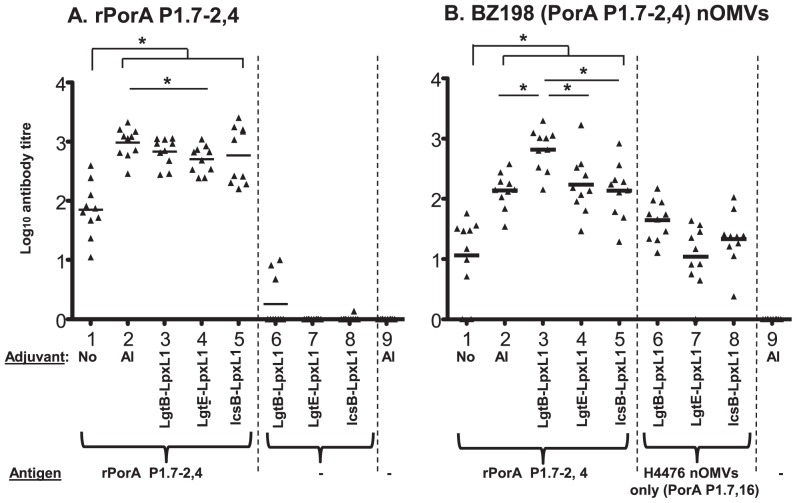
ELISA against (A) rPorA P1.7-2,4 and (B) BZ198 OMVs. Mouse groups 1-5 received 5 µg rPorA P1.7-2,4 adjuvanted with the following: 1) no adjuvant, 2) Alhydrogel, 3) H44/76-LgtB-LpxL1 nOMV, 4) H44/76-LgtE-LpxL1 nOMV, 5) H44/76-IcsB-LpxL1 nOMV. Groups 6-9 did not contain any rPorA and were immunized with the following nOMVs: 6) H44/76-LgtB-LpxL1, 7) H44/76-LgtE-LpxL1, 8) H44/76-IcsB-LpxL1. Group 9 received Alhydrogel alone. Mice were immunized twice at 4 and 8 weeks and serum samples were taken 2 weeks after the last injection. Each symbol represents an individual animal; the horizontal bar represents the mean of the group. Statistical differences observed between groups containing rPorA are noted on the graphs (*: p<0.05). Statistical differences between rPorA-immunized groups of mice and control groups (nOMVs only or no antigen) are not shown.

The mouse experiment was performed once, with 10 animals per group, but power calculation indicated that this number of animals would provide 99.9% statistical power at an Alpha error level of 5% to detect a 2-fold difference with a two-tail test. Sera from immunized mice were tested in an ELISA designed to detect the concentration of anti-rPorA P1.7-2,4 antibodies. As expected, immunization with rPorA alone elicited a modest anti-PorA response, which was significantly increased by addition of Alhydrogel adjuvant (Fig. 5A, lanes 1 and 2). All three nOMVs elicited serum IgG responses (Fig. 5A, lanes 3–5), and there were no significant differences in antibody titres between Alhydrogel and nOMVs from strains H44/76-LgtB-LpxL1 or H44/76-IcsB-LpxL1. Only the H44/76-LgtE-LpxL1 nOMVs induced lower antibodies when compared to Alhydrogel (*p*<*0.05*, Fig. 5A lanes 2 and 4). The concentration of IgG antibodies specific to rPorA P1.7-2,4 was low or undetectable in mice immunized with the nOMVs alone (nOMVs contain PorA P1.7, 16, Fig. 5, lanes 6–8). This is in agreement with previous reports suggesting that the PorA-specific responses elicited by OMVs essentially target the hyper-variable regions VR1 and VR2 [32], [33].

### The three glycoform nOMVs variants induce antibody responses to BZ198 nOMV expressing PorA P1.7-2,4

Strain BZ198 expresses the same PorA type (P1.7-2,4) as the rPorA used in the murine immunizations. As predicted, sera from mice immunized with the recombinant rPorA protein recognized native PorA P1.7-2,4 within the BZ198 nOMVs. Sera from all the mice immunized with H44/76 nOMVs and rPorA recognized BZ198 nOMVs to varying degrees (Fig. 5B, lanes 3–5). Differences were observed in the adjuvant activity of the three H44/76-derived nOMVs with respect to BZ198 nOMVs: nOMVs from H44/76-LgtB-LpxL1 raised significantly greater concentrations of antibodies than nOMVs from H44/76-LgtE-LpxL1 (*p*<*0.05*), H44/76-IcsB-LpxL1 (*p*<*0.05*), and Alhydrogel (*p*<*0.05*) (Fig. 5B, lanes 2–5). In contrast, H44/76-IcsB-LpxL1nOMVs and H44/76-LgtE-lpxL1 nOMVs each showed no differences in adjuvant effects compared to Alhydrogel. Of note, control groups of mice immunized with H44/76 nOMVs alone (containing PorA P1.7,16) and in the absence of rPorA P1.7-2,4 (lanes 6–9) elicited low IgG responses against BZ198 nOMVs. A possible explanation for these findings is that cross-reactive antibodies targeting PorA are elicited or antigens other than PorA, that are conserved between strains H44/76 and BZ198, raise cross-reacting antibodies.

### The truncated glycoforms present in H44/76-LgtB-Lpxl1 and H44/76-LgtE-Lpxl1 nOMVs induce bactericidal activity against BZ198 strain

SBA activity was not detected in mice immunized with rPorA alone (titres <1∶4, Table 3). The use of Alhydrogel or nOMVs from H44/76-IcsB-LpxL1 in the formulation did not result in significant levels of bactericidal activity (titres <1∶4 and <1∶8 respectively). However, sera from mice immunized with rPorA mixed with nOMVs from strains H44/76-LgtB-LpxL1 and H44/76-LgtE-LpxL1 elicited SBA titres of 1∶128 and 1∶64 respectively (a mean of two determinations with pooled sera, Table 3).

**Table 3:** Bactericidal titres of pooled murine sera obtained after immunisation with rPorA P1.7-2,4 adjuvated with Alhydrogel or various nOMVs.

Group	Antigen	Adjuvant	Adjuvant dose (µg)	Bactericidal titer
1	rPorA	-		<1∶4
2	rPorA	Alhydrogel	33	<1∶4
3	rPorA	nOMV: H44/76-LgtB-LpxL1	2.5	1∶128
4	rPorA	nOMV: H44/76-IcsB-LpxL1	2.5	<1∶8
5	rPorA	nOMV: H44/76-LgtE-LpxL1	2.5	1∶64
6		nOMV: H44/76-LgtB-LpxL1	2.5	<1∶4
7		nOMV: H44/76-IcsB-LpxL1	2.5	<1∶4
8		nOMV: H44/76-LgtE-LpxL1	2.5	<1∶4
9		Alhydrogel	330	<1∶4

Titres are expressed as the reciprocal of the last dilution at which ≥50% killing was achieved.

## Discussion

Our study demonstrates that each of the LPS structures investigated (H44/76-LgtB-LpxL1, H44/76-LgtE-LpxL1 and H44/76-IcsB-LpxL1), when delivered in nOMVs, increased the immunogenicity of purified rPorA P1.7-2,4 as measured by serum IgG concentrations in mice. In contrast to other formulations, H44/76-LgtB-LpxL1 and to a lower extent H44/76-LgtE-LpxL1 resulted in antibodies with bactericidal activity. Thus this study provides *in vivo* confirmation of the proposed adjuvant properties of LgtB-LpxL1 LPS [14], [16], [34]. However, SBA titers measured with baby rabbit complement are higher than observed with human complement due to the species-specificity of the meningococcal factor H binding protein. Therefore it is not known whether this adjuvant effect would be observed in humans. In addition, not all adjuvant properties can be attributed to LPS given nOMVs contain a multitude of other components. Other antigens, such as PorB are known to possess adjuvant properties [35], [36], [37]}. However, all other antigens present on H44/76 nOMVs should be equivalent as each of the nOMVs used in the experiment was derived from a set of isogenic strains.

Since LgtB-Lpxl1 nOMVs induce lower IL-6 production than IcSB-Lpxl1 and LgtE-Lpxl1 in a human blood assay, it seems likely that the higher SBA titers induced by this mutant as compared to the other two nOMVs is not related to higher reactogenicity of the former glycoform. The adjuvant properties observed in the nOMVs may be explained through the interaction of LPS with the LpxL1 phenotype (LpxL1 LPS) with murine TLR4, probably inducing release of inflammatory cytokines such as TNF, IL-6 and IL-1, and maturation of DCs [38], [39]. Murine studies have shown that LpxL1 LPS can restore the adjuvant properties of LPS-deficient outer membrane complexes as measured by ELISA and SBA [9]. The mediation of these effects through TLR4 has also been confirmed *in vivo* with TLR4-deficient mice [40], [41]. Disrupting the *lpxL1* gene allows safe administration of relatively large doses of LPS in humans [7]. As nOMVs from *lpxL1* mutants contain five times as much LPS than deoxycholate-extracted OMVs (dOMVs) [6], greater quantities of the oligosaccharide core of LPS can be exposed to the immune system whilst maintaining safety [42].

Studies *in vitro* have shown that LPS oligosaccharide moieties bind effectively with dendritic-cell-specific ICAM-3 grabbing non-integrin (DC-SIGN) lectin receptors present on human immature DCs. Activation of DC-SIGN results in ligand internalization and downstream signalling that serves to skew T-cell responses toward T helper type 1 activity [15], [16]. DCs have been shown to be more easily activated by meningococcal strain H44/76 expressing truncated LPS with the LgtB phenotype (LgtB LPS) in comparison to fully extended LPS [15], [16]. A limitation to understanding the details of the mechanism underlying our results is that no receptor identical to DC-SIGN has yet been identified on murine DCs. The functional ortholog of DC-SIGN in mice has been labelled mDC-SIGN. mDC-SIGN differs appreciably in its ligand-binding profile and only shares 57% peptide sequence homology with DC-SIGN [43], [44]. However, our results do suggest that there exists a murine receptor that is capable of discriminating between specific LPS oligosaccharide structures, as demonstrated by differences in the antibody titres and bactericidal activities obtained.

TLR4 and DC-SIGN homologues interact in both mice and humans. In humans, the DC-SIGN signalling cascade converges with TLR4 signalling at the level of NFκB to modulate the cytokines released by DCs [45], [46]. These cytokines influence the maturation of naïve T helper cells towards a Th1 or Th2 response [47]. In mice, it has been shown that mSIGN-R1, another murine ortholog of DC-SIGN, recognizes the oligosaccharide core of LPS, in particular GlcNAc-Glc-Gal in *Salmonella* species [48], [49], [50]. Activation of mSIGN-R1 modulates downstream signal transduction and subsequent pro-inflammatory cytokine production through association with the TLR4-MD2 complex [50]. In our study, we observed different adjuvant properties between the various oligosaccharide structures, but the adjuvant action of nOMVs expressing various LPS penta-acylated glycoforms cannot be easily dissected.

There must inevitably be caution exerted in the extrapolation of results derived from a murine model to humans. It has been observed that human TLR4 does not respond to LpxL1 LPS as potently as murine TLR4 [34], [51]. Although this suggests that penta-acylated LPS would be even less endotoxic in humans, the adjuvant benefits of using penta-acylated LPS may also be diminished. On the other hand, the trisaccharide GlcNAc-Gal-Glc resembles the outer core of LgtB LPS, making it an excellent ligand for human DC-SIGN, one know to play an important role in modulating immune responses.
